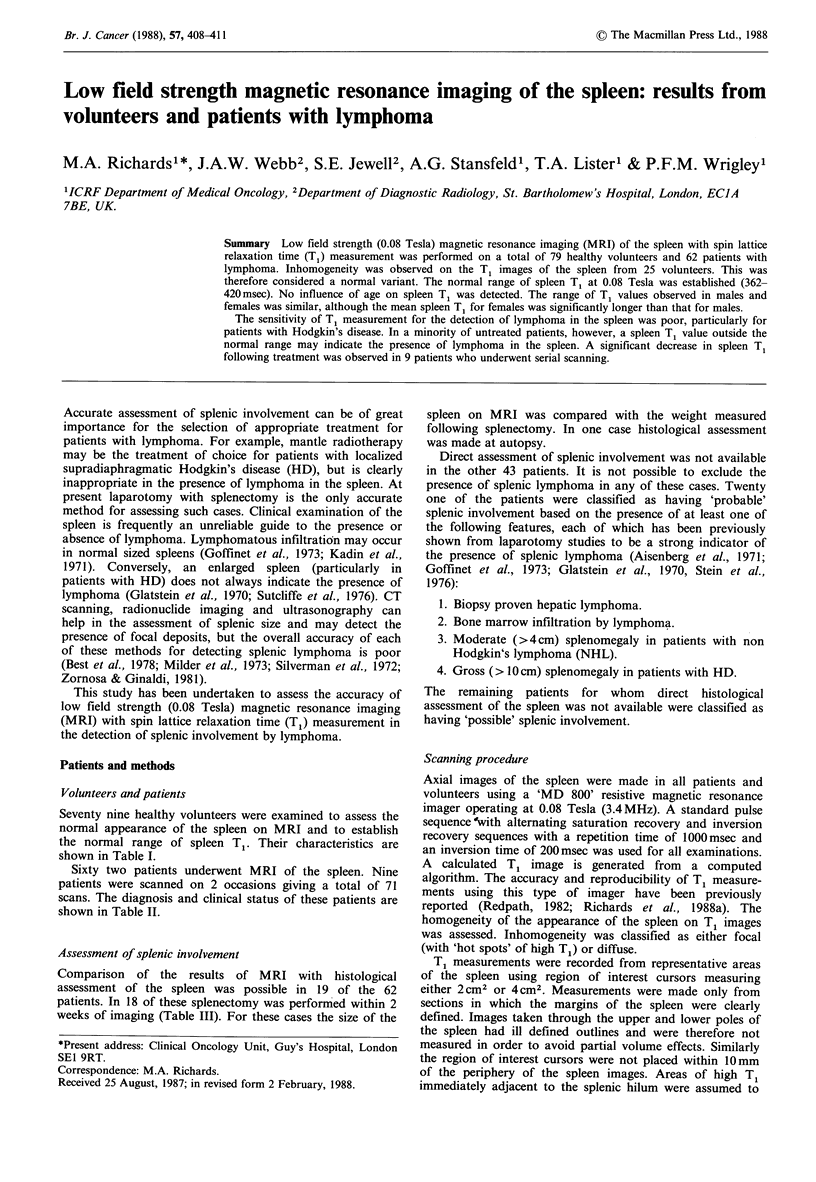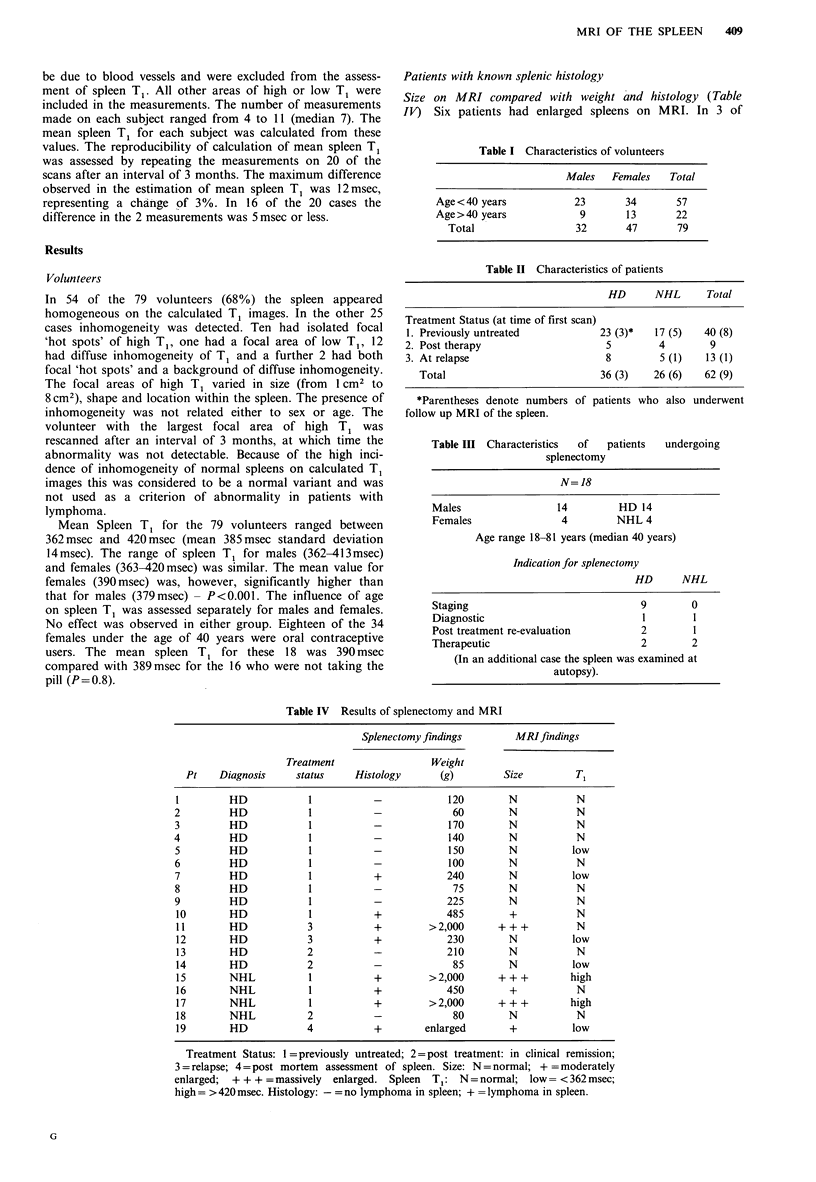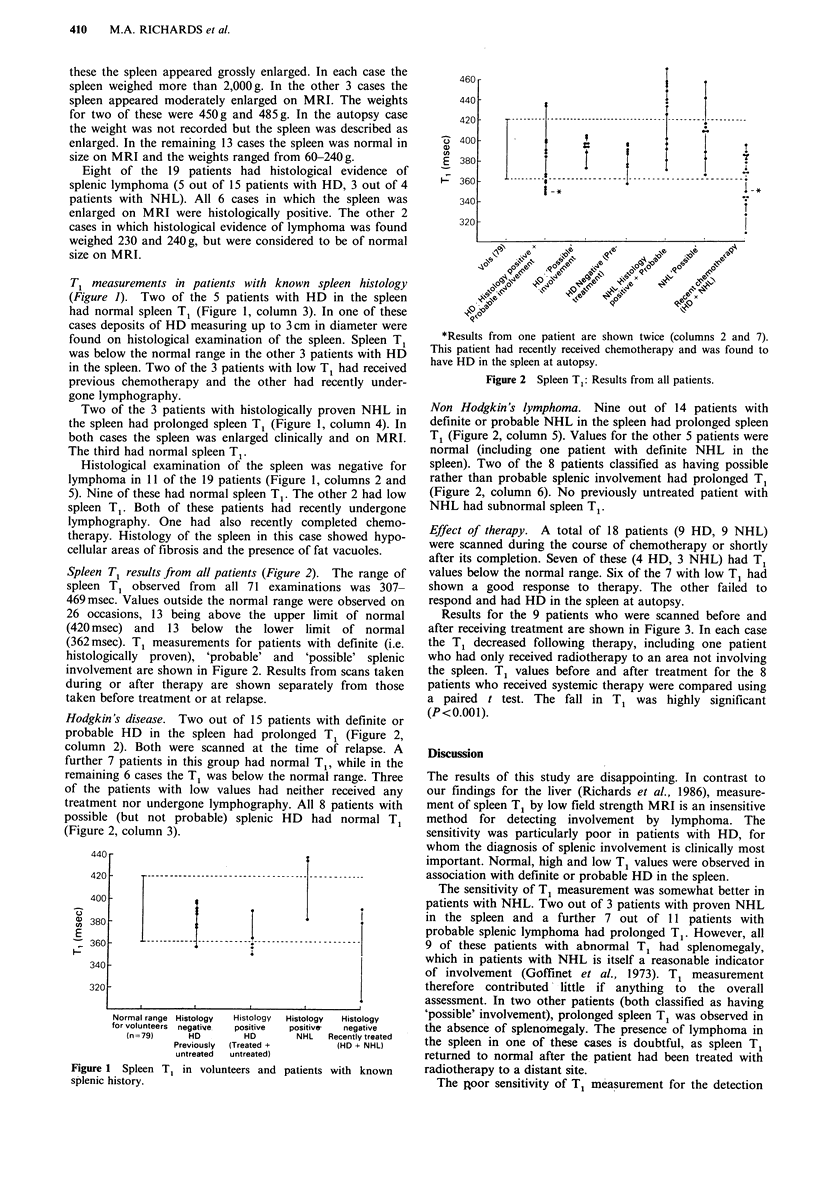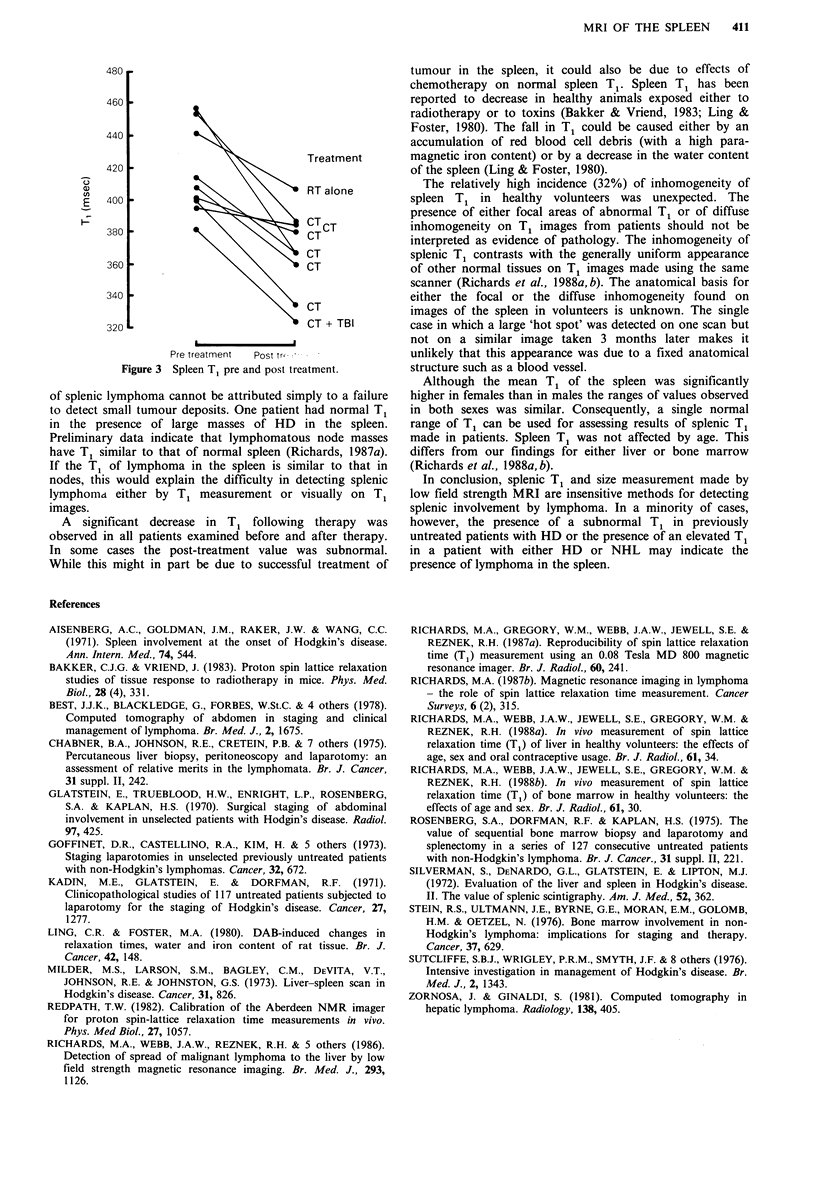# Low field strength magnetic resonance imaging of the spleen: results from volunteers and patients with lymphoma.

**DOI:** 10.1038/bjc.1988.92

**Published:** 1988-04

**Authors:** M. A. Richards, J. A. Webb, S. E. Jewell, A. G. Stansfeld, T. A. Lister, P. F. Wrigley

**Affiliations:** ICRF Department of Medical Oncology, St. Bartholomew's Hospital, London, UK.

## Abstract

Low field strength (0.08 Tesla) magnetic resonance imaging (MRI) of the spleen with spin lattice relaxation time (T1) measurement was performed on a total of 79 healthy volunteers and 62 patients with lymphoma. Inhomogeneity was observed on the T1 images of the spleen from 25 volunteers. This was therefore considered a normal variant. The normal range of spleen T1 at 0.08 Tesla was established (362-420 msec). No influence of age on spleen T1 was detected. The range of T1 values observed in males and females was similar, although the mean spleen T1 for females was significantly longer than that for males. The sensitivity of T1 measurement for the detection of lymphoma in the spleen was poor, particularly for patients with Hodgkin's disease. In a minority of untreated patients, however, a spleen T1 value outside the normal range may indicate the presence of lymphoma in the spleen. A significant decrease in spleen T1 following treatment was observed in 9 patients who underwent serial scanning.


Br. J. Cancer (1988), 57, 408–411 © The Macmillan Press Ltd., 1988

### Low field strength magnetic resonance imaging of the spleen: results from volunteers and patients with lymphoma

M.A. Richards[1]*, J.A.W. Webb[2], S.E. Jewell[2], A.G. Stansfeld[1], T.A. Lister[1] & P.F.M. Wrigley[1]

[1]ICRF Department of Medical Oncology, [2]Department of Diagnostic Radiology, St. Bartholomew's Hospital, London, EC1A 7BE, UK.

**Summary** Low field strength (0.08 Tesla) magnetic resonance imaging (MRI) of the spleen with spin lattice relaxation time ($T_1$) measurement was performed on a total of 79 healthy volunteers and 62 patients with lymphoma. Inhomogeneity was observed on the $T_1$ images of the spleen from 25 volunteers. This was therefore considered a normal variant. The normal range of spleen $T_1$ at 0.08 Tesla was established (362–420 msec). No influence of age on spleen $T_1$ was detected. The range of $T_1$ values observed in males and females was similar, although the mean spleen $T_1$ for females was significantly longer than that for males.

The sensitivity of $T_1$ measurement for the detection of lymphoma in the spleen was poor, particularly for patients with Hodgkin's disease. In a minority of untreated patients, however, a spleen $T_1$ value outside the normal range may indicate the presence of lymphoma in the spleen. A significant decrease in spleen $T_1$ following treatment was observed in 9 patients who underwent serial scanning.

Accurate assessment of splenic involvement can be of great importance for the selection of appropriate treatment for patients with lymphoma. For example, mantle radiotherapy may be the treatment of choice for patients with localized supradiaphragmatic Hodgkin's disease (HD), but is clearly inappropriate in the presence of lymphoma in the spleen. At present laparotomy with splenectomy is the only accurate method for assessing such cases. Clinical examination of the spleen is frequently an unreliable guide to the presence or absence of lymphoma. Lymphomatous infiltration may occur in normal sized spleens (Goffinet *et al.*, 1973; Kadin *et al.*, 1971). Conversely, an enlarged spleen (particularly in patients with HD) does not always indicate the presence of lymphoma (Glatstein *et al.*, 1970; Sutcliffe *et al.*, 1976). CT scanning, radionuclide imaging and ultrasonography can help in the assessment of splenic size and may detect the presence of focal deposits, but the overall accuracy of each of these methods for detecting splenic lymphoma is poor (Best *et al.*, 1978; Milder *et al.*, 1973; Silverman *et al.*, 1972; Zornosa & Ginaldi, 1981).

This study has been undertaken to assess the accuracy of low field strength (0.08 Tesla) magnetic resonance imaging (MRI) with spin lattice relaxation time ($T_1$) measurement in the detection of splenic involvement by lymphoma.

### Patients and methods

### *Volunteers and patients*

Seventy nine healthy volunteers were examined to assess the normal appearance of the spleen on MRI and to establish the normal range of spleen $T_1$. Their characteristics are shown in Table I.

Sixty two patients underwent MRI of the spleen. Nine patients were scanned on 2 occasions giving a total of 71 scans. The diagnosis and clinical status of these patients are shown in Table II.

### *Assessment of splenic involvement*

Comparison of the results of MRI with histological assessment of the spleen was possible in 19 of the 62 patients. In 18 of these splenectomy was performed within 2 weeks of imaging (Table III). For these cases the size of the spleen on MRI was compared with the weight measured following splenectomy. In one case histological assessment was made at autopsy.

Direct assessment of splenic involvement was not available in the other 43 patients. It is not possible to exclude the presence of splenic lymphoma in any of these cases. Twenty one of the patients were classified as having 'probable' splenic involvement based on the presence of at least one of the following features, each of which has been previously shown from laparotomy studies to be a strong indicator of the presence of splenic lymphoma (Aisenberg *et al.*, 1971; Goffinet *et al.*, 1973; Glatstein *et al.*, 1970, Stein *et al.*, 1976):

1. Biopsy proven hepatic lymphoma.
2. Bone marrow infiltration by lymphoma.
3. Moderate (>4 cm) splenomegaly in patients with non Hodgkin's lymphoma (NHL).
4. Gross (>10 cm) splenomegaly in patients with HD.

The remaining patients for whom direct histological assessment of the spleen was not available were classified as having 'possible' splenic involvement.

### *Scanning procedure*

Axial images of the spleen were made in all patients and volunteers using a 'MD 800' resistive magnetic resonance imager operating at 0.08 Tesla (3.4 MHz). A standard pulse sequence with alternating saturation recovery and inversion recovery sequences with a repetition time of 1000 msec and an inversion time of 200 msec was used for all examinations. A calculated $T_1$ image is generated from a computed algorithm. The accuracy and reproducibility of $T_1$ measurements using this type of imager have been previously reported (Redpath, 1982; Richards *et al.*, 1988a). The homogeneity of the appearance of the spleen on $T_1$ images was assessed. Inhomogeneity was classified as either focal (with 'hot spots' of high $T_1$) or diffuse.

$T_1$ measurements were recorded from representative areas of the spleen using region of interest cursors measuring either 2 cm² or 4 cm². Measurements were made only from sections in which the margins of the spleen were clearly defined. Images taken through the upper and lower poles of the spleen had ill defined outlines and were therefore not measured in order to avoid partial volume effects. Similarly the region of interest cursors were not placed within 10 mm of the periphery of the spleen images. Areas of high $T_1$ immediately adjacent to the splenic hilum were assumed to

*Present address: Clinical Oncology Unit, Guy's Hospital, London SE1 9RT.
Correspondence: M.A. Richards.
Received 25 August, 1987; in revised form 2 February, 1988.

be due to blood vessels and were excluded from the assessment of spleen $T_1$. All other areas of high or low $T_1$ were included in the measurements. The number of measurements made on each subject ranged from 4 to 11 (median 7). The mean spleen $T_1$ for each subject was calculated from these values. The reproducibility of calculation of mean spleen $T_1$ was assessed by repeating the measurements on 20 of the scans after an interval of 3 months. The maximum difference observed in the estimation of mean spleen $T_1$ was 12 msec, representing a change of 3%. In 16 of the 20 cases the difference in the 2 measurements was 5 msec or less.

### Results

### *Volunteers*

In 54 of the 79 volunteers (68%) the spleen appeared homogeneous on the calculated $T_1$ images. In the other 25 cases inhomogeneity was detected. Ten had isolated focal 'hot spots' of high $T_1$, one had a focal area of low $T_1$, 12 had diffuse inhomogeneity of $T_1$ and a further 2 had both focal 'hot spots' and a background of diffuse inhomogeneity. The focal areas of high $T_1$ varied in size (from 1 $cm^2$ to 8 $cm^2$), shape and location within the spleen. The presence of inhomogeneity was not related either to sex or age. The volunteer with the largest focal area of high $T_1$ was rescanned after an interval of 3 months, at which time the abnormality was not detectable. Because of the high incidence of inhomogeneity of normal spleens on calculated $T_1$ images this was considered to be a normal variant and was not used as a criterion of abnormality in patients with lymphoma.

Mean Spleen $T_1$ for the 79 volunteers ranged between 362 msec and 420 msec (mean 385 msec standard deviation 14 msec). The range of spleen $T_1$ for males (362–413 msec) and females (363–420 msec) was similar. The mean value for females (390 msec) was, however, significantly higher than that for males (379 msec) – $P<0.001$. The influence of age on spleen $T_1$ was assessed separately for males and females. No effect was observed in either group. Eighteen of the 34 females under the age of 40 years were oral contraceptive users. The mean spleen $T_1$ for these 18 was 390 msec compared with 389 msec for the 16 who were not taking the pill ($P=0.8$).

### *Patients with known splenic histology*

*Size on MRI compared with weight and histology (Table IV)* Six patients had enlarged spleens on MRI. In 3 of

**Table I** Characteristics of volunteers

| | *Males* | *Females* | *Total* |
|---|---|---|---|
| Age < 40 years | 23 | 34 | 57 |
| Age > 40 years | 9 | 13 | 22 |
| Total | 32 | 47 | 79 |

**Table II** Characteristics of patients

| | *HD* | *NHL* | *Total* |
|---|---|---|---|
| Treatment Status (at time of first scan) | | | |
| 1. Previously untreated | 23 (3)* | 17 (5) | 40 (8) |
| 2. Post therapy | 5 | 4 | 9 |
| 3. At relapse | 8 | 5 (1) | 13 (1) |
| Total | 36 (3) | 26 (6) | 62 (9) |

*Parentheses denote numbers of patients who also underwent follow up MRI of the spleen.

**Table III** Characteristics of patients undergoing splenectomy

| *N=18* | | |
|---|---|---|
| Males | 14 | HD 14 |
| Females | 4 | NHL 4 |

Age range 18–81 years (median 40 years)

*Indication for splenectomy*

| | *HD* | *NHL* |
|---|---|---|
| Staging | 9 | 0 |
| Diagnostic | 1 | 1 |
| Post treatment re-evaluation | 2 | 1 |
| Therapeutic | 2 | 2 |

(In an additional case the spleen was examined at autopsy).

**Table IV** Results of splenectomy and MRI

| | | | *Splenectomy findings* | | *MRI findings* | |
|---|---|---|---|---|---|---|
| *Pt* | *Diagnosis* | *Treatment status* | *Histology* | *Weight (g)* | *Size* | $T_1$ |
| 1 | HD | 1 | – | 120 | N | N |
| 2 | HD | 1 | – | 60 | N | N |
| 3 | HD | 1 | – | 170 | N | N |
| 4 | HD | 1 | – | 140 | N | N |
| 5 | HD | 1 | – | 150 | N | low |
| 6 | HD | 1 | – | 100 | N | N |
| 7 | HD | 1 | + | 240 | N | low |
| 8 | HD | 1 | – | 75 | N | N |
| 9 | HD | 1 | – | 225 | N | N |
| 10 | HD | 1 | + | 485 | + | N |
| 11 | HD | 3 | + | >2,000 | +++ | N |
| 12 | HD | 3 | + | 230 | N | low |
| 13 | HD | 2 | – | 210 | N | N |
| 14 | HD | 2 | – | 85 | N | low |
| 15 | NHL | 1 | + | >2,000 | +++ | high |
| 16 | NHL | 1 | + | 450 | + | N |
| 17 | NHL | 1 | + | >2,000 | +++ | high |
| 18 | NHL | 2 | – | 80 | N | N |
| 19 | HD | 4 | + | enlarged | + | low |

Treatment Status: 1 = previously untreated; 2 = post treatment: in clinical remission; 3 = relapse; 4 = post mortem assessment of spleen. Size: N = normal; + = moderately enlarged; +++ = massively enlarged. Spleen $T_1$: N = normal; low = <362 msec; high = >420 msec. Histology: — = no lymphoma in spleen; + = lymphoma in spleen.

these the spleen appeared grossly enlarged. In each case the spleen weighed more than 2,000 g. In the other 3 cases the spleen appeared moderately enlarged on MRI. The weights for two of these were 450 g and 485 g. In the autopsy case the weight was not recorded but the spleen was described as enlarged. In the remaining 13 cases the spleen was normal in size on MRI and the weights ranged from 60–240 g.

Eight of the 19 patients had histological evidence of splenic lymphoma (5 out of 15 patients with HD, 3 out of 4 patients with NHL). All 6 cases in which the spleen was enlarged on MRI were histologically positive. The other 2 cases in which histological evidence of lymphoma was found weighed 230 and 240 g, but were considered to be of normal size on MRI.

*$T_1$ measurements in patients with known spleen histology (Figure 1).* Two of the 5 patients with HD in the spleen had normal spleen $T_1$ (Figure 1, column 3). In one of these cases deposits of HD measuring up to 3 cm in diameter were found on histological examination of the spleen. Spleen $T_1$ was below the normal range in the other 3 patients with HD in the spleen. Two of the 3 patients with low $T_1$ had received previous chemotherapy and the other had recently undergone lymphography.

Two of the 3 patients with histologically proven NHL in the spleen had prolonged spleen $T_1$ (Figure 1, column 4). In both cases the spleen was enlarged clinically and on MRI. The third had normal spleen $T_1$.

Histological examination of the spleen was negative for lymphoma in 11 of the 19 patients (Figure 1, columns 2 and 5). Nine of these had normal spleen $T_1$. The other 2 had low spleen $T_1$. Both of these patients had recently undergone lymphography. One had also recently completed chemotherapy. Histology of the spleen in this case showed hypocellular areas of fibrosis and the presence of fat vacuoles.

*Spleen $T_1$ results from all patients (Figure 2).* The range of spleen $T_1$ observed from all 71 examinations was 307–469 msec. Values outside the normal range were observed on 26 occasions, 13 being above the upper limit of normal (420 msec) and 13 below the lower limit of normal (362 msec). $T_1$ measurements for patients with definite (i.e. histologically proven), 'probable' and 'possible' splenic involvement are shown in Figure 2. Results from scans taken during or after therapy are shown separately from those taken before treatment or at relapse.

*Hodgkin's disease.* Two out of 15 patients with definite or probable HD in the spleen had prolonged $T_1$ (Figure 2, column 2). Both were scanned at the time of relapse. A further 7 patients in this group had normal $T_1$, while in the remaining 6 cases the $T_1$ was below the normal range. Three of the patients with low values had neither received any treatment nor undergone lymphography. All 8 patients with possible (but not probable) splenic HD had normal $T_1$ (Figure 2, column 3).

**Figure 1** Spleen $T_1$ in volunteers and patients with known splenic history.

*Results from one patient are shown twice (columns 2 and 7). This patient had recently received chemotherapy and was found to have HD in the spleen at autopsy.

**Figure 2** Spleen $T_1$: Results from all patients.

*Non Hodgkin's lymphoma.* Nine out of 14 patients with definite or probable NHL in the spleen had prolonged spleen $T_1$ (Figure 2, column 5). Values for the other 5 patients were normal (including one patient with definite NHL in the spleen). Two of the 8 patients classified as having possible rather than probable splenic involvement had prolonged $T_1$ (Figure 2, column 6). No previously untreated patient with NHL had subnormal spleen $T_1$.

*Effect of therapy.* A total of 18 patients (9 HD, 9 NHL) were scanned during the course of chemotherapy or shortly after its completion. Seven of these (4 HD, 3 NHL) had $T_1$ values below the normal range. Six of the 7 with low $T_1$ had shown a good response to therapy. The other failed to respond and had HD in the spleen at autopsy.

Results for the 9 patients who were scanned before and after receiving treatment are shown in Figure 3. In each case the $T_1$ decreased following therapy, including one patient who had only received radiotherapy to an area not involving the spleen. $T_1$ values before and after treatment for the 8 patients who received systemic therapy were compared using a paired $t$ test. The fall in $T_1$ was highly significant ($P<0.001$).

### Discussion

The results of this study are disappointing. In contrast to our findings for the liver (Richards et al., 1986), measurement of spleen $T_1$ by low field strength MRI is an insensitive method for detecting involvement by lymphoma. The sensitivity was particularly poor in patients with HD, for whom the diagnosis of splenic involvement is clinically most important. Normal, high and low $T_1$ values were observed in association with definite or probable HD in the spleen.

The sensitivity of $T_1$ measurement was somewhat better in patients with NHL. Two out of 3 patients with proven NHL in the spleen and a further 7 out of 11 patients with probable splenic lymphoma had prolonged $T_1$. However, all 9 of these patients with abnormal $T_1$ had splenomegaly, which in patients with NHL is itself a reasonable indicator of involvement (Goffinet et al., 1973). $T_1$ measurement therefore contributed little if anything to the overall assessment. In two other patients (both classified as having 'possible' involvement), prolonged spleen $T_1$ was observed in the absence of splenomegaly. The presence of lymphoma in the spleen in one of these cases is doubtful, as spleen $T_1$ returned to normal after the patient had been treated with radiotherapy to a distant site.

The poor sensitivity of $T_1$ measurement for the detection

**Figure 3** Spleen $T_1$ pre and post treatment.

of splenic lymphoma cannot be attributed simply to a failure to detect small tumour deposits. One patient had normal $T_1$ in the presence of large masses of HD in the spleen. Preliminary data indicate that lymphomatous node masses have $T_1$ similar to that of normal spleen (Richards, 1987*a*). If the $T_1$ of lymphoma in the spleen is similar to that in nodes, this would explain the difficulty in detecting splenic lymphoma either by $T_1$ measurement or visually on $T_1$ images.

A significant decrease in $T_1$ following therapy was observed in all patients examined before and after therapy. In some cases the post-treatment value was subnormal. While this might in part be due to successful treatment of tumour in the spleen, it could also be due to effects of chemotherapy on normal spleen $T_1$. Spleen $T_1$ has been reported to decrease in healthy animals exposed either to radiotherapy or to toxins (Bakker & Vriend, 1983; Ling & Foster, 1980). The fall in $T_1$ could be caused either by an accumulation of red blood cell debris (with a high paramagnetic iron content) or by a decrease in the water content of the spleen (Ling & Foster, 1980).

The relatively high incidence (32%) of inhomogeneity of spleen $T_1$ in healthy volunteers was unexpected. The presence of either focal areas of abnormal $T_1$ or of diffuse inhomogeneity on $T_1$ images from patients should not be interpreted as evidence of pathology. The inhomogeneity of splenic $T_1$ contrasts with the generally uniform appearance of other normal tissues on $T_1$ images made using the same scanner (Richards *et al.*, 1988*a,b*). The anatomical basis for either the focal or the diffuse inhomogeneity found on images of the spleen in volunteers is unknown. The single case in which a large 'hot spot' was detected on one scan but not on a similar image taken 3 months later makes it unlikely that this appearance was due to a fixed anatomical structure such as a blood vessel.

Although the mean $T_1$ of the spleen was significantly higher in females than in males the ranges of values observed in both sexes was similar. Consequently, a single normal range of $T_1$ can be used for assessing results of splenic $T_1$ made in patients. Spleen $T_1$ was not affected by age. This differs from our findings for either liver or bone marrow (Richards *et al.*, 1988*a,b*).

In conclusion, splenic $T_1$ and size measurement made by low field strength MRI are insensitive methods for detecting splenic involvement by lymphoma. In a minority of cases, however, the presence of a subnormal $T_1$ in previously untreated patients with HD or the presence of an elevated $T_1$ in a patient with either HD or NHL may indicate the presence of lymphoma in the spleen.

### References

AISENBERG, A.C., GOLDMAN, J.M., RAKER, J.W. & WANG, C.C. (1971). Spleen involvement at the onset of Hodgkin's disease. *Ann. Intern. Med.*, **74**, 544.

BAKKER, C.J.G. & VRIEND, J. (1983). Proton spin lattice relaxation studies of tissue response to radiotherapy in mice. *Phys. Med. Biol.*, **28** (4), 331.

BEST, J.J.K., BLACKLEDGE, G., FORBES, W.St.C. & 4 others (1978). Computed tomography of abdomen in staging and clinical management of lymphoma. *Br. Med. J.*, **2**, 1675.

CHABNER, B.A., JOHNSON, R.E., CRETEIN, P.B. & 7 others (1975). Percutaneous liver biopsy, peritoneoscopy and laparotomy: an assessment of relative merits in the lymphomata. *Br. J. Cancer*, **31** suppl. II, 242.

GLATSTEIN, E., TRUEBLOOD, H.W., ENRIGHT, L.P., ROSENBERG, S.A. & KAPLAN, H.S. (1970). Surgical staging of abdominal involvement in unselected patients with Hodgin's disease. *Radiol.* **97**, 425.

GOFFINET, D.R., CASTELLINO, R.A., KIM, H. & 5 others (1973). Staging laparotomies in unselected previously untreated patients with non-Hodgkin's lymphomas. *Cancer*, **32**, 672.

KADIN, M.E., GLATSTEIN, E. & DORFMAN, R.F. (1971). Clinicopathological studies of 117 untreated patients subjected to laparotomy for the staging of Hodgkin's disease. *Cancer*, **27**, 1277.

LING, C.R. & FOSTER, M.A. (1980). DAB-induced changes in relaxation times, water and iron content of rat tissue. *Br. J. Cancer*, **42**, 148.

MILDER, M.S., LARSON, S.M., BAGLEY, C.M., DEVITA, V.T., JOHNSON, R.E. & JOHNSTON, G.S. (1973). Liver–spleen scan in Hodgkin's disease. *Cancer*, **31**, 826.

REDPATH, T.W. (1982). Calibration of the Aberdeen NMR imager for proton spin-lattice relaxation time measurements *in vivo*. *Phys. Med Biol.*, **27**, 1057.

RICHARDS, M.A., WEBB, J.A.W., REZNEK, R.H. & 5 others (1986). Detection of spread of malignant lymphoma to the liver by low field strength magnetic resonance imaging. *Br. Med. J.*, **293**, 1126.

RICHARDS, M.A., GREGORY, W.M., WEBB, J.A.W., JEWELL, S.E. & REZNEK, R.H. (1987*a*). Reproducibility of spin lattice relaxation time ($T_1$) measurement using an 0.08 Tesla MD 800 magnetic resonance imager. *Br. J. Radiol.*, **60**, 241.

RICHARDS, M.A. (1987*b*). Magnetic resonance imaging in lymphoma – the role of spin lattice relaxation time measurement. *Cancer Surveys*, **6** (2), 315.

RICHARDS, M.A., WEBB, J.A.W., JEWELL, S.E., GREGORY, W.M. & REZNEK, R.H. (1988*a*). *In vivo* measurement of spin lattice relaxation time ($T_1$) of liver in healthy volunteers: the effects of age, sex and oral contraceptive usage. *Br. J. Radiol.*, **61**, 34.

RICHARDS, M.A., WEBB, J.A.W., JEWELL, S.E., GREGORY, W.M. & REZNEK, R.H. (1988*b*). *In vivo* measurement of spin lattice relaxation time ($T_1$) of bone marrow in healthy volunteers: the effects of age and sex. *Br. J. Radiol.*, **61**, 30.

ROSENBERG, S.A., DORFMAN, R.F. & KAPLAN, H.S. (1975). The value of sequential bone marrow biopsy and laparotomy and splenectomy in a series of 127 consecutive untreated patients with non-Hodgkin's lymphoma. *Br. J. Cancer.*, **31** suppl. II, 221.

SILVERMAN, S., DENARDO, G.L., GLATSTEIN, E. & LIPTON, M.J. (1972). Evaluation of the liver and spleen in Hodgkin's disease. II. The value of splenic scintigraphy. *Am. J. Med.*, **52**, 362.

STEIN, R.S., ULTMANN, J.E., BYRNE, G.E., MORAN, E.M., GOLOMB, H.M. & OETZEL, N. (1976). Bone marrow involvement in non-Hodgkin's lymphoma: implications for staging and therapy. *Cancer*, **37**, 629.

SUTCLIFFE, S.B.J., WRIGLEY, P.R.M., SMYTH, J.F. & 8 others (1976). Intensive investigation in management of Hodgkin's disease. *Br. Med. J.*, **2**, 1343.

ZORNOSA, J. & GINALDI, S. (1981). Computed tomography in hepatic lymphoma. *Radiology*, **138**, 405.